# Progress in State Medicine

**Published:** 1899-09-30

**Authors:** 


### Progress in State Medicine.

### HOUSING OF THE WORKING CLASSES.

The questions of overcrowding, the destruction of slum property, and the proper housing of the working-class are closely associated. There is still a steady immigration of the rural population into the manufacturing towns, and this, together with the high price of land in the cities, and the constant destruction of insanitary areas, has rendered the problem of housing one of the most difficult and pressing of the day. In spite, however, of the legal difficulties and enormous expense incurred in grappling with this problem, improvement schemes have been or are being carried through in many of the large towns, such as London, Edinburgh, Liverpool, Dublin, Manchester, and Southampton.

The Housing of the Working-Classes Act of 1890 consists of three parts. Part I. deals with the repair, closing, or demolition of individual houses, the demolition of obstructive buildings, and of small areas of unhealthy houses. Part II. refers to improvement schemes in connection with large areas or slums; and Part III. to the erection of lodging-houses for the working classes. Evans[1] points out that the procedure under this Act is cumbersome, and that in many towns, such as Liverpool and Manchester, local Acts are found preferable. The chief obstacles to the working of the Act are the inability of councillors to judge of sanitary conditions, the fear of expense, or of interfering with private rights. Owing to legal delays four or five years may pass before progress of the scheme can be reported. He has successfully dealt with an area in Bradford under Part I. It consisted of 284 houses, with a density of population equal to 301 persons per acre. The death-rate from all causes averaged 43·1 per 1,000, and the phthisis death-rate was 6·8 per 1,000.

Addie[2] recommends that in all cases local authorities framing an improvement scheme should first of all make provision for the displaced population, for the Local Government Board always insist upon this point. In planning streets both the main and side streets should be wide, and awkwardly-shaped plots, difficult to build upon, should be avoided. It is more profitable for the local authorities to let the ground at a ground rent than to build themselves. His experience of improvement schemes at Swansea and Birmingham under local powers has been favourable. In the former place a very undesirable area of 14 acres was cleared at a cost of £120,000. The Birmingham scheme dealt with an area of 45 acres (1878-1882), with 1,867 houses and 814 other buildings. The total net expenditure has been £1,686,148. It is calculated that the loans will be paid off in 44 years, and a net rental income of £53,000 per annum carried to the credit of the City funds. Speaking of the houses erected to accommodate displaced workmen, the writer states that 22 through cottages cost £4,000, and are let at 5s. 6d. per week. A further 81 dwellings cost £14,000, and let for 5s. to 6s. per week. Each cottage consisted of a living-room, 13 ft. by 12 ft. 6 in.; kitchen, 11 ft. 6 in. by 9 ft. 4 in.; front bed-room, 13 ft. by 12 ft. 6 in.; back bed-room, 12 ft. 6 in. by 9 ft. 4 in.; landing and attic, 13 ft. 3 in. by 12 ft. 6 in.; a separate w.c. for each family, washhouses and concrete yard common to each block. Some new cottages now in course of erection have the following dimensions: Living-room, 13 ft. 4 in. by 14 ft.; bed-room, 8 ft. 2 in. by 14 ft.; bed-room, 9 ft. by 9 ft. The rents have been fixed at 1s. 6d. per week per room. Each tenement will be provided with a separate w.c., scullery accommodation, and ash receptacle. Sixty-one houses will cost £11,025. The types of dwellings now being erected are: In Dublin, five-storey flats, dual houses, and one-storey cottages; in Liverpool, five and six-storey flats, but latterly, both in this town and in Manchester, dual houses; in London, chiefly flats.

The importance of providing for family life with due regard to the separation of the sexes is emphasised by Sykes.[3] Investigations in Glasgow show that there is an increasing tendency for working families to gravitate to three or four-room houses, abandoning larger houses, and one-room tenements. In England the same tendency is evident, but a larger proportion live in one-room tenements. In block dwellings passages must have free access to the open air; there must be no back-to-back buildings, water-closets and bath-houses should be separated from the dwelling quarters, and the space at front and rear of the building should be as wide as the height of the house. No dwelling-room should have an area of less than 96 ft., and the minimum height should be 9 ft. The cubic space per head for adults should be 400 ft., and this should be fixed by statutory definition.

The manufacture of new slum property is a subject dealt with by Newsholme.[4] He points out that the jerry-built houses of to-day are not calculated to stand for a long period, and when unsafe may be condemned under Part II. of the Housing of Working Classes Act. A more serious danger is the building over or covering in of back yards. This procedure renders through ventilation impracticable, and alone is sufficient to render the house unfit for human habitation. The legal procedure is, however, difficult in such cases, and unless the new building be discovered within six months it cannot be removed. The evil might be met by increasing the surveyor's staff, or by the surveyor's office co-operating with the sanitary and water inspectors.

The better housing of the poor is to a great extent a financial problem.[5] It has been shown that in the poorer parts of London 88 per cent. of the population pay more than a fifth of their income in rent, and 46 per cent. pay from one-fourth to one half. Even in houses built and let by municipal authorities the cost per room is high, 3s. to 4s. in London, 1s. 6d. in Birmingham, and 1s. in Liverpool. The last census showed that tenements generally were much overcrowded. In towns over 12 per cent. of the population live in such an overcrowded condition that even when all living rooms are counted as bedrooms, there are still more than two persons sleeping in each room. The great cause of overcrowding is high rental, brought about by the greater expense of building materials and labour in recent years. The demolition of insanitary areas is a most expensive process. In London two areas were cleared, and the cost per family amount to £300 and £500 respectively. In Brighton the cost per person in two areas was £27 12s. 9d. and £30 15s. With regard to rehousing, it is essential that this should precede demolition, so that the value of the insanitary property may be lowered, and the actual persons displaced may be housed. In London and other large towns the erection of blocks, such as the Peabody and Guinness buildings, seems absolutely unavoidable. The general health of the inhabitants is good, the death-rate being below the average, except for whooping-cough and measles. They require the strictest supervision. In Liverpool they have succeeded in building tenements, let at 2s. 6d. per week, consisting of two rooms and a scullery. These are built in three-storey blocks. If the Local Government Board would allow the repayment of loans to be spread over 60 instead of 30 years it would enable local authorities to let their tenements at far lower rates. Even an extension of 10 years, such as they recently granted to one local body, enabled them to reduce the weekly rent of a six-roomed cottage from 8s. 6d. to 7s. 3d. per week. With regard to municipal lodging-houses, these, although fairly successful, have not yielded such good financial results or been so popular as private enterprises such as Rowton House. The Bristol Corporation have, during the present year, adopted Part III. of the Housing of the Working Classes Act, and purchased for £1,562 a site of 588 square yards, on which, for a sum of £5,150 (exclusive of furniture) they propose erecting a lodging-house to contain 102 beds.

Fyfe[6] gives the following calculation with regard to the expense of workmen's dwellings. With ground at 30s. a square yard (3 per cent. interest) a four-storey block dwelling may be built at a cost of 4½d. per cubic foot of contents, providing a sanitary and comfortable house of 2,000 cubic feet at a weekly rent of 2s. 5½d. This is on the presumption that the money is borrowed at 2¾ per cent., and allowance is made for depreciation and contingencies.

[1] Public Health, April, 1899. [2] Jour. San. Instit., April, 1899. [3] Jour. San. Instit., April, 1899. [4] Public Health, Aug., 1899. [5] Practitioner, July and Aug., 1899. [6] Lecture: Housing of the Labouring Classes.

